# Efficacy of pazopanib and sunitinib in advanced axial chordoma: a single reference centre case series

**DOI:** 10.1186/s13569-016-0059-x

**Published:** 2016-11-01

**Authors:** Astrid Lipplaa, Sander Dijkstra, Hans Gelderblom

**Affiliations:** 1 Department of Medical Oncology, Leiden University Medical Centre, Albinusdreef 2, Leiden, The Netherlands; 2 Department of Orthopaedic Surgery, Leiden University Medical Centre, Albinusdreef 2, Leiden, The Netherlands

**Keywords:** Chordoma, Vascular endothelial growth factor, Tyrosine kinase inhibitor, Pazopanib, Sunitinib

## Abstract

**Background:**

Chordomas are rare malignant tumours of the axial skeleton and skull base supposed to arise from cellular remnants of the notochord. These tumours have the potential to metastasize (30–40 %), usually in the later course of the disease. However, the greatest morbidity is usually a result of loco-regional recurrence with infiltration and destruction of surrounding bone and soft tissue. Patients with unresectable or metastatic chordoma are faced with a poor prognosis since cytotoxic chemotherapy or other systemic therapies have not proven their efficacy yet. However, several molecularly targeted drugs have been proposed as potentially beneficial, including tyrosine kinase inhibitors (TKIs) directed at vascular endothelial growth factor receptor (VEGFR), like pazopanib and sunitinib.

**Case presentation:**

Five patients with unresectable or metastatic chordoma were treated with VEGFR inhibitors pazopanib or sunitinib in the Leiden University Medical Centre (LUMC) between 2008 and 2015. Two out of four patients treated with pazopanib derived clinical benefit and disease remained stable for respectively 14 and 15 months. The one patient treated with sunitinib achieved a partial response according to RECIST 1.1 which lasted for a total of 27 months. No serious adverse events were observed.

**Conclusion:**

These results on the use of pazopanib and sunitinib in chordoma are promising, with an objective response on sunitinib and a median progression free interval of 8.5 months (range 3–15 months), comparable to that of imatinib, in the pazopanib subgroup. However further research is needed to assess the definite role of VEGFR inhibitors in chordoma.

## Background

Chordomas are rare malignant bone tumours of the axial skeleton and skull base that have a prevalence of less than 1/100,000 [1]. They are supposed to arise from cellular remnants of the notochord that persist during foetal development [2]. These tumours have the potential to metastasize (30–40 %), usually in the later course of the disease. However, the greatest morbidity is generally a result of loco-regional recurrence with infiltration and destruction of surrounding bone and soft tissue [2–4].

The first consensus guidelines for the diagnosis and treatment of chordoma were published in the Lancet Oncology in 2015 [4]. In patients with localized disease, complete surgical resection with adequate margins offers the best chance of long-term control. Definitive (proton beam) radiotherapy is the preferred alternative in case resection is not feasible. The addition of standard adjuvant radiotherapy is recommended for skull base and cervical spine chordoma. In sacral chordoma the consensus guidelines recommend consideration of radiotherapy in case of R1 resection [4]. However, results from another retrospective patient series show that the time to recurrence is significantly longer with the addition of radiotherapy, accompanied by a trend towards longer overall survival, and thus support the strategy to add radiotherapy as standard adjuvant therapy [5]. Ten-year local recurrence free survival rates after initial surgery is 33–49 %. In case of loco-regional recurrence prognosis is poor and a number of palliative treatment modalities can be considered, e.g. (debulking) surgery and (stereotactic or heavy particle) radiation [4].

Cytotoxic chemotherapy or other systemic therapies have not proven their efficacy and are therefore not recommended in any line of treatment [4]. A better molecular understanding of chordoma can be helpful to identify targetable pathways. So far, only systemic treatment with the platelet derived growth factor receptor (PDGFR) tyrosine kinase inhibitor (TKI) imatinib has shown positive results in a phase 2 study in advanced chordoma. This trial included 56 patients of whom one (2 %) achieved a partial response according to RECIST 1.1 and another 20 % a minor response. Median progression-free survival was 9 months. Moreover, 64 % of patients derived clinical benefit and 70 % of patients stable disease (SD) [6]. The positive results of this trial have led to funding for its use in chordoma patients in some countries.

Several other molecularly targeted drugs directed at pathways believed to be implicated in chordoma have been evaluated, including the epidermal growth factor receptor (EGFR)-inhibitors erlotinib, gefitinib and lapatinib, the mTOR (mechanistic target of rapamycin)-inhibitor sirolimus, and vascular endothelial growth factor (VEGF)-inhibitor bevacizumab. Most of these drugs have shown modest activity in case reports and case series, administered either as monotherapy or combined with other agents [7–13]. A dedicated phase 2 study of lapatinib in EGFR-positive chordoma resulted in a progression free survival (PFS) of 8 months [14]. Furthermore, a phase 2 study on sorafenib in 27 patients with advanced and metastatic chordoma by the French Sarcoma Group, resulted in a 9-month progression free rate of 73 % and a 12-month overall survival rate of 86.5 % [15].

The effect of pazopanib and sunitinib was only published for a limited numbers of patients (pazopanib 1 patient and sunitinib 9 patients) [16, 17]. These multi-targeted kinase inhibitors not only inhibit PDFGR, but also the vascular endothelial growth factor receptor (VEGFR) considered relevant for chordoma progression.

Inhibition of pro-angiogenic growth factors like VEGFR may be relevant in chordoma genesis and proliferation. Increased expression of established angiogenesis-related factors VEGF, hypoxia-inducible factor-1α (HIF-1α) and matrix metalloproteinase (MMP)-2 and -9 is observed in chordoma tissue and cell lines [18, 19]. Moreover, expression of MMP-9 was associated with an increased local recurrence rate and unfavourable prognosis [19]. These findings highlight the important role of angiogenesis in chordoma, and VEGFR inhibitors could therefore be a promising therapeutic drug class.

This consecutive case series reports the response of chordoma patients to VEGFR TKIs pazopanib and sunitinib. In the Leiden University Medical Centre, a total of five chordoma patients were treated with these agents between 2008 and 2015; four patients with pazopanib and one patient with sunitinib. The patients treated with pazopanib were part of the “TIP-study”, a prospective pharmacological study investigating the feasibility of pharmacokinetics (PK)-guided individualized dosing to reduce the interpatient variability in pazopanib exposure [20].

## Cases

See Table 1 for an overview of patients, tumour characteristics and results.

**Table 1:** Overview patients treated with pazopanib and sunitinib

Pt	Localization	Prior local Tx	Prior systemic Tx	Drug/dose	Results (RECIST 1.1 criteria)	Adverse events
1	Sacrum	Intralesional excision + cryosurgery Adjuvant radiotherapy	–	Pazopanib 600 mg/day	SD 14 months Symptomatic improvement	Rash gr 1, intermittent diarrhoea max gr 2, fatigue gr 2, loss pigment hair
2	Sacrum	–	–	Pazopanib 800 mg/day	SD 15 months Symptomatic improvement	Fatigue gr 1
3	Sacrum	En-bloc resection (adequate margins)	–	Pazopanib 800 mg/day	PD 3 months	Intermittent nausea and vomiting gr 1
4	Thoracic spine	Intralesional excision 3x Stereotactic radiotherapy after 1st surgery	Imatinib	Pazopanib 800 mg/day	PD 3 months, paraplegia	–
5	Lumbar spine	Intralesional excision 3x Adjuvant radiotherapy after 2nd surgery	Imatinib	Sunitinib 37.5 mg/day	Radiologic response (PR) 3 months SD 27 months Symptomatic improvement	Dose reduction due to gr 2 nausea. Other symptoms: intermittent fatigue gr 2, hand-foot syndrome gr 1, epistaxis gr 1, thrombocytopenia gr 2

All pathology samples were reviewed and/or revised by pathologists at the LUMC/Dutch Committee on Bone Tumours. IHC staining for brachyury was only performed (and found to be positive) on the tumour sample for patient 1. Diagnosis was based on microscopy findings and IHC staining for pankeratin, keratin AE1/3, vimentin, S100 and/or MIB-1 for all patients

The first patient is a 47-year old male who was referred to our hospital for systemic treatment of a multifocal recurrence of his sacral chordoma. The chordoma was diagnosed 2 years earlier and treated with intralesional excision and cryosurgery, followed by postoperative radiotherapy (60 Gy, boost of 66 Gy presacral area of suspected irradical resection). Pazopanib was started at a dose of 800 mg/day, and reduced to 600 mg/day 4 weeks later due to blood area under the curve (AUC) levels above the minimum threshold for activity [20, 21]. Overall, pazopanib was well tolerated; the patient reported loss of hair pigment, grade 1 skin rash on his legs, fatigue grade 2 and intermittent diarrhoea (maximum grade 2) for which he successfully used loperamide. The disease remained stable according to RECIST 1.1 (with scans approximately every 4 months) for 14 months until the patient noticed increasing back pain radiating to his left buttock. MRI scan showed progression of the sacral tumour mass. Since other tumour localizations remained stable, pazopanib was continued for another 6 months while a palliative sacral resection was scheduled. The patient died of progressive disease 9 months after resection and discontinuation of pazopanib.

**Fig. 1:**
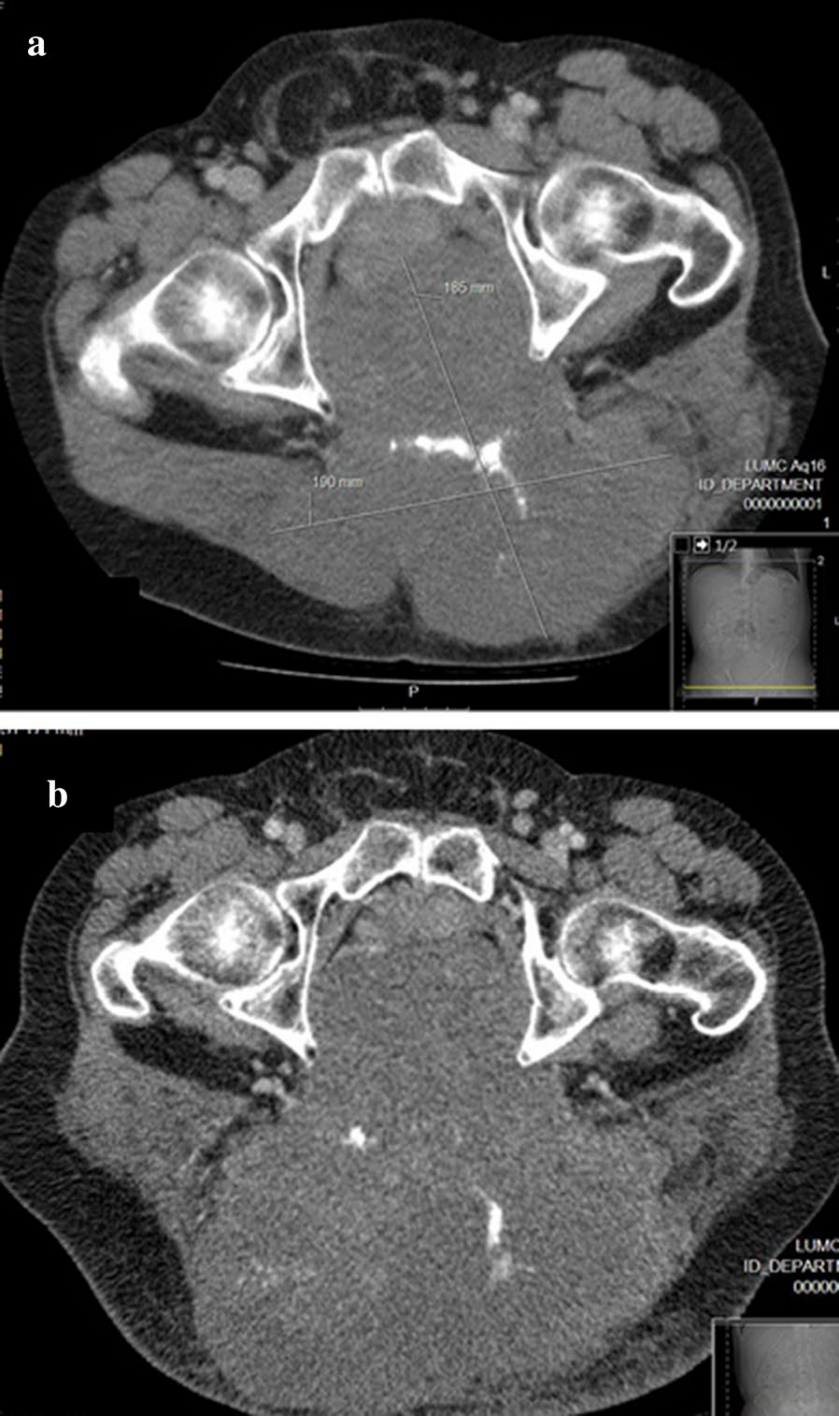
CT images of patient 2 treated with pazopanib. **a** CT scan at start of pazopanib showing a large sacral chordoma reaching from S2 to coccygis. Measurements: 20 × 16 × 13 cm (AP × LR × CC). **b** CT scan after 15 months of pazopanib showing progressive disease. Measurements: 21 × 24 × 23 cm (AP × LR × CC)

Patient 2 is a 54-year old male who presented with coccygodynia since a fall on his tail bone 10 years before. Initial imaging showed a chordoma of the sacrum with a maximum diameter of 18 cm, compressing the rectum and bladder (see Fig. 1a). Since the tumour had a close relationship to bladder, rectum and skin, resection was not deemed feasible and the patient was started on systemic treatment with pazopanib (800 mg/day). After 4 weeks of treatment the patient reported symptomatic improvement and he could taper his analgesic medication. Apart from grade 1 fatigue, no other pazopanib related side-effects were reported. CT scans at 4, 6 and 10 months of treatment showed stable disease according to RECIST 1.1. After 15 months however, there were clinical and radiological signs of progression (maximum diameter of 24 cm, see Fig. 1b). After extensive discussion, patient declined en-bloc resection due to significant predicted morbidity. Since pazopanib was tolerated very well, treatment was continued for a total of 27 months until evident local disease progression. The patient chose a further palliative treatment course because of rapid deterioration of his condition, and he died 2 months later.

Patient 3 is a 66-year old male with a sacral chordoma diagnosed in 2010. Primary treatment, consisting of en-bloc resection with adequate margins, was complicated by post-operative urosepsis and development of a large seroma. Scheduled adjuvant radiotherapy was omitted due to these complications. A multifocal local recurrence was diagnosed 2 years later and first line systemic treatment with pazopanib (800 mg/day) started. The patient reported intermittent grade 1 nausea, but tolerated treatment overall well. Unfortunately, progressive disease according to RECIST 1.1 was seen on MRI after only 3 months of treatment. Pazopanib was discontinued and patient went on to have palliative radiotherapy on subcutaneous metastases (36 Gy). His disease is clinically stable since radiotherapy treatment in March 2013, although MRI scans do show progression of existing lesions.

The fourth and last patient treated with pazopanib is a 42-year old male with a chordoma of the spine, primary lesion in corpus of Th11 with associated myelum compression. He was diagnosed 3 years before and underwent three consecutive intralesional resections with stereotactic radiotherapy (39 Gy) after the first surgery. He presented in our hospital with progressive intraspinal and hepatic metastases after 9 months of systemic treatment with imatinib. Second line treatment with pazopanib was started at a dose of 800 mg/day, and patient was temporarily dose reduced to 600 mg/day due to elevated AUC levels. Aside from grade 1 haemoptysis, related to an incidental pulmonary embolism, no other side-effects were reported. Three months after the start of pazopanib, this patient was hospitalized with acute paraplegia secondary to progressive spinal lesions (RECIST 1.1 progressive disease). Pazopanib was discontinued and there were no further surgical or radiotherapy options for this patient. He died a year later as a result of disease progression.

**Fig. 2:**
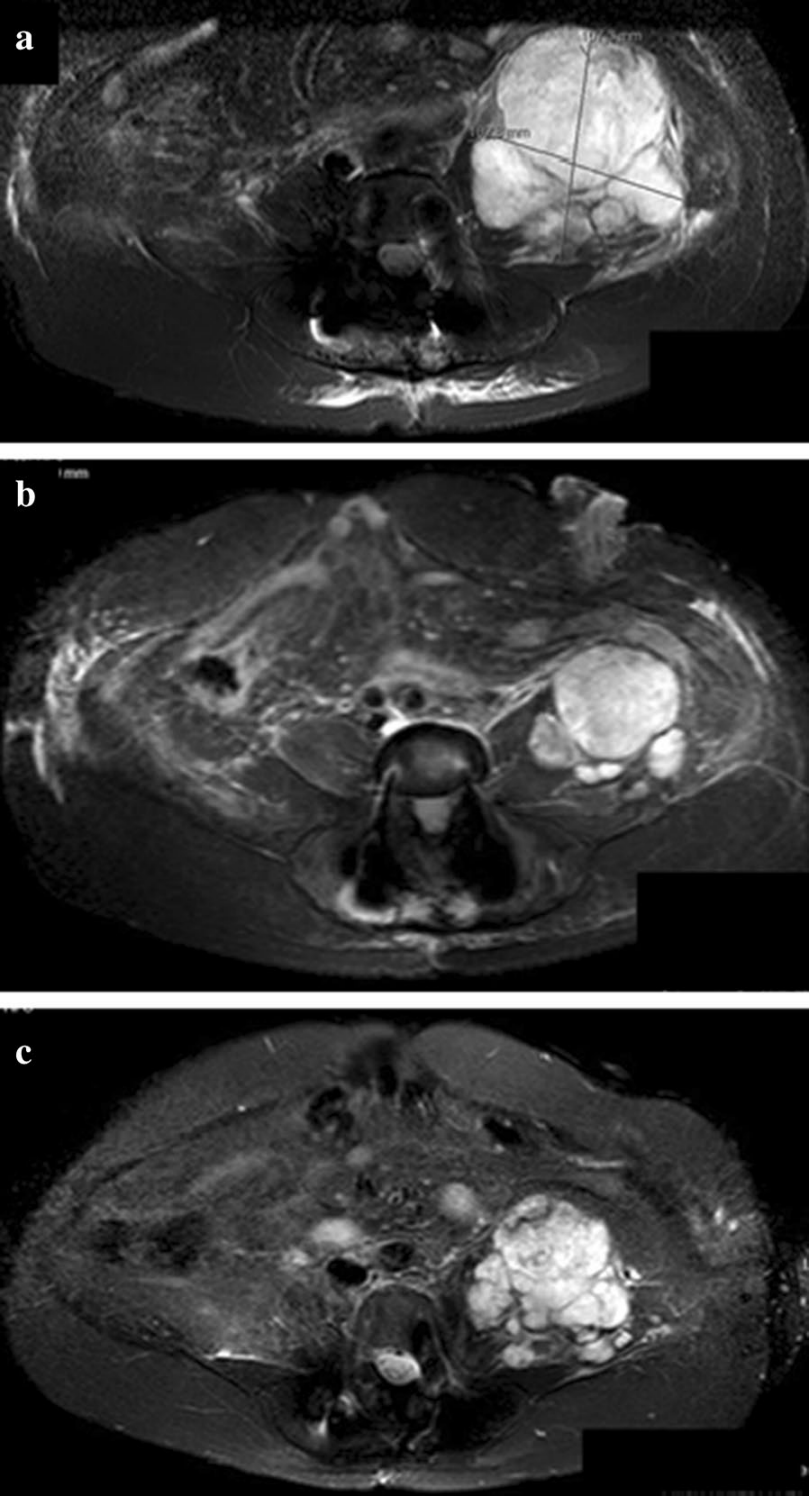
MRI images (T2 gadolinium and fat suppression) of patient 5 treated with sunitinib. **a** MRI scan at start of sunitinib showing a large retroperitoneal metastasis of a primary lumbar spine chordoma. Measurements: 10.3 × 10.7 × 9.4 cm (LR × AP × CC). **b** MRI scan after 6 months of sunitinib showing a partial response according to RECIST 1.1. Measurements: 9.5 × 7.4 × 8.3 cm (LR × AP × CC). **c** MRI scan showing progressive disease after 27 months of treatment. Measurements: 8.9 × 10 cm (LR × AP)

The fifth patient, a 59-year old female with a chordoma of corpus L1, was treated with sunitinib. She underwent three intralesional resections, adjuvant radiotherapy (60 Gy) and systemic treatment with imatinib during 6 months until progression. The MRI scan before the start of sunitinib treatment showed a large retroperitoneal metastasis with a maximum diameter of 11 cm (anterior posterior [AP] measurement, see Fig. 2a). Sunitinib was commenced at a dose of 50 mg/day (4 weeks on, 2 weeks off), and lowered to 37.5 mg/day due to on-going grade 2 nausea. Other reported symptoms include: intermittent grade 2 fatigue, grade 1 hand-foot syndrome, grade 1 epistaxis and grade 2 thrombocytopenia. Follow-up MRI scans after 3 and 6 months showed a partial response according to RECIST 1.1 within the retroperitoneal lesion, shrinking down to maximum AP diameter of 7.5 cm (Fig. 2b). Further stabilization of disease lasted for 27 months. On progression, the patient was switched to imatinib and sirolimus combination treatment, which resulted in further tumour growth after 4 months (Fig. 2c). The patient died due to progressive disease 18 months later.

Progression-free interval was defined as time from first dose of drug until date of confirmed radiological disease progression according to RECIST 1.1. All patients reached the RECIST 1.1 progressive disease endpoint. The mean progression-free interval for patients in the pazopanib group was 8.5 months and 14 months overall.

## Discussion

This case series describes the effect of VEGFR TKIs pazopanib in four patients and sunitinib in one patient with unresectable or progressive chordoma treated in the Leiden University Medical Centre.

A partial response according to RECIST 1.1 was seen in the only patient using sunitinib and three out of five patients reported symptomatic improvement. The median progression free interval was 8.5 months in the pazopanib subgroup, 27 months in the patient treated with sunitinib and 14 months overall. Treatment was well tolerated with only mild to moderate side effects. In the pazopanib group patients reported the following side effects: skin rash (25 %), fatigue (50 %), diarrhoea (25 %) and nausea (25 %). The patient on sunitinib was treated with a reduced dose due to on-going grade 2 nausea and experienced fatigue, hand-foot syndrome and thrombocytopenia. The dose reductions in the pazopanib patients were solely based on blood drug levels as a part of the TIP study and were not a result of adverse events.

Side effects seen in our patients were expected and comparable to those seen in other populations [16, 22]. No serious adverse events were observed.

Limitations to this case series are primarily its retrospective and unplanned analysis and small patient numbers. Response evaluation was not standardized as imaging was performed every 2–6 months for the different patients.

However, results from small case series like this are important, since experience with the use of pazopanib and sunitinib in chordoma in literature reports is limited. A phase 2 trial of sunitinib in 53 patients with nongastrointestinal stromal tumour sarcomas included nine patients with chordoma. No radiological responses by RECIST 1.1 or metabolic responses by FDG-PET were seen, but four out of nine chordoma patients had stable disease for a period ranging from 17 to 70 weeks [16]. We could find only one Japanese case report on pazopanib used in chordoma. This patient achieved a partial response with a significant reduction in tumour size and was treated for a total of 14 months until progression [17].

## Conclusion

The results from our case series on the use of VEGFR inhibitors pazopanib and sunitinib in chordoma are promising, with an partial response according to RECIST 1.1 on sunitinib and a median PFS of 8.5 months, comparable to that of imatinib [6], in the pazopanib subgroup. New treatment strategies are urgently needed for these patients with unresectable chordomas: a situation that can lead to severe morbidity and represents an unmet medical need. Large randomized trials will not be feasible due to the rarity of the disease. A definite role of VEGFR TKIs can only be established after a prospective phase II study, either randomized cross-over or compared to historical controls aiming for at least 12 months PFS in first line or 9 months in second of later line after imatinib. We would like to challenge regulators, pharmaceutical industry and sponsors to support such registration trials with VEGFR TKIs in this difficult disease state.

## Abbreviations

AUC: area under the curve
EGFR: epidermal growth factor receptor
HIF-1α: hypoxia-inducible factor 1 alpha
MMP: matrix metalloproteinase
mTOR: mammalian target of rapamycin
PDGFR: platelet derived growth factor receptor
PFS: progression free survival
PK: pharmacokinetic
SD: stable disease
TKI: tyrosine kinase inhibitor
VEGFR: vascular endothelial growth factor receptor
